# CERS6 required for cell migration and metastasis in lung cancer

**DOI:** 10.1111/jcmm.15817

**Published:** 2020-09-09

**Authors:** Motoshi Suzuki, Ke Cao, Seiichi Kato, Naoki Mizutani, Kouji Tanaka, Chinatsu Arima, Mei Chee Tai, Norie Nakatani, Kiyoshi Yanagisawa, Toshiyuki Takeuchi, Hanxiao Shi, Yasuyoshi Mizutani, Atsuko Niimi, Tetsuo Taniguchi, Takayuki Fukui, Kohei Yokoi, Keiko Wakahara, Yoshinori Hasegawa, Yukiko Mizutani, Soichiro Iwaki, Satoshi Fujii, Akira Satou, Keiko Tamiya‐Koizumi, Takashi Murate, Mamoru Kyogashima, Shuta Tomida, Takashi Takahashi

**Affiliations:** ^1^ Division of Molecular Carcinogenesis Nagoya University Graduate School of Medicine Nagoya Japan; ^2^ Department of Molecular Oncology Fujita Health University Toyoake Japan; ^3^ Department of Pathology and Laboratory Medicine Nagoya University Hospital Nagoya Japan; ^4^ Department of Medical Technology Nagoya University Graduate School of Health Sciences Nagoya Japan; ^5^ Department of Thoracic Surgery Nagoya University Graduate School of Medicine Nagoya Japan; ^6^ Department of Respiratory Medicine Nagoya University Graduate School of Medicine Nagoya Japan; ^7^ Laboratory of Biomembrane and Biofunctional Chemistry Faculty of Advanced Life Science Hokkaido University Sapporo Japan; ^8^ Department of Molecular and Cellular Pathobiology and Therapeutics Graduate School of Pharmaceutical Sciences Nagoya City University Nagoya Japan; ^9^ Division of Microbiology and Molecular Cell Biology Nihon Pharmaceutical University Saitama Japan; ^10^ Department of Biobank Okayama University Graduate School of Medicine Dentistry and Pharmaceutical Sciences Okayama Japan; ^11^ Present address: Department of Oncology The Third Xiangya Hospital of Central South University Changsha China; ^12^ Present address: Satoshi Fujii Department of Laboratory Medicine Asahikawa Medical University Asahikawa Japan; ^13^ Present address: Department of Surgical Pathology Aichi Medical University Hospital Nagakute Japan

**Keywords:** ceramide, lung cancer, metastasis, micro‐RNA

## Abstract

Sphingolipids constitute a class of bio‐reactive molecules that transmit signals and exhibit a variety of physical properties in various cell types, though their functions in cancer pathogenesis have yet to be elucidated. Analyses of gene expression profiles of clinical specimens and a panel of cell lines revealed that the ceramide synthase gene *CERS6* was overexpressed in non–small‐cell lung cancer (NSCLC) tissues, while elevated expression was shown to be associated with poor prognosis and lymph node metastasis. NSCLC profile and *in vitro* luciferase analysis results suggested that *CERS6* overexpression is promoted, at least in part, by reduced *miR‐101* expression. Under a reduced CERS6 expression condition, the ceramide profile became altered, which was determined to be associated with decreased cell migration and invasion activities *in vitro*. Furthermore, CERS6 knockdown suppressed RAC1‐positive lamellipodia/ruffling formation and attenuated lung metastasis efficiency in mice, while forced expression of CERS6 resulted in an opposite phenotype in examined cell lines. Based on these findings, we consider that ceramide synthesis by CERS6 has important roles in lung cancer migration and metastasis.

## 1 INTRODUCTION

Accumulating evidence indicates that bio‐reactive ceramides, which comprise a family of molecules with a variety of acyl chains and structures, and their metabolic enzymes may play roles in cancer.^1^ It has also been shown that they transmit signals under various stress conditions, such as ionizing radiation, cytokine exposure and therapeutic agent administration.^2^ Other reports noted that ceramides including d18:1‐C16:0 ceramide (C16 ceramide) serve as apoptosis mediators in response to TNF‐α^3^ or other pro‐apoptotic stimuli,^4^ while it was also demonstrated that, as compared to normal tissue levels, ceramide amounts were significantly increased in human head and neck squamous cell carcinoma (HNSCC).^5^ In addition to ceramides, ceramide synthases (CERSs) as well are elevated in breast cancer tissues.^6^ Moreover, ceramides are required for survival of some HNSCC cells, while ceramide synthase 6 (CERS6) suppression was found to be associated with induction of ER stress and apoptosis.^7^ Together, these findings suggest that CERSs and ceramides are required for cancer cell phenotypes, whereas their contribution mechanisms to carcinogenesis have yet to be elucidated.

Lung cancer is the leading cause of cancer deaths in many countries. In order to reduce this intolerable death toll, studies to understand the molecular mechanisms of cancer initiation and progression, as well as cancer‐specific metabolic pathways, are required to begin development of novel treatments. Among the various approaches to achieve that goal, we have undertaken examinations of gene expression profiles of lung cancer specimens and identified pathways and genes that contribute to cancer pathogenesis.^8^, ^9^, ^10^, ^11^

Here, we analysed sphingolipid metabolic genes regarding their lung cancer‐associated expression profiles, which revealed CERS6 as a pivotal protein for lung cancer progression.

## 2 METHODS

### 2.1 Cell lines and materials

The ACC‐LC‐176 and NCI‐H460‐LNM35 (LNM35) cell lines were previously reported.^12^ Cancer cells were cultured in RPMI 1640 supplemented with 5 or 10% FBS (Gibco). The immortalized lung epithelial cell line BEAS‐2B was maintained as previously described.^13^ All were tested and confirmed to be free from mycoplasma contamination. An anti‐CERS6 antibody was purchased from Abnova (clone 5H7), anti‐RAC1 from Millipore (clone23A8), anti‐α‐tubulin from Sigma‐Aldrich, anti‐ceramide from Glycobiotech (S58‐9) and anti‐PKCζ and anti‐pPKCζ from Santa Cruz (SC216 and SC12894‐R, respectively). Anti‐mouse IgG and anti‐rabbit IgG were conjugated with HRP from Cell Signaling Technology. Anti‐mouse IgG and anti‐rabbit IgG conjugated with Alexa 488 or 568 were from Invitrogen. Fumonisin B_1_ (Cayman Chemical) and myriocin (Sigma‐Aldrich) were also purchased. Sequence information of the oligonucleotide primers used for PCR and sequencing is provided in Table S1.

### 2.2 Oligonucleotides and transfection

For transfection of LNM35, 10 nM of small interfering RNA (siRNA) duplex (Sigma‐Aldrich) targeting *CERS* (Table S1), or corresponding concentration of Negative Control (Mission siRNA Universal Negative Control #2, Sigma), 1 nM of the pre‐miR miRNA Precursor Molecule of *miR‐101* or Negative Control #2 (both from Applied Biosystems) were used with Lipofectamine™ RNAiMax (Invitrogen). BEAS‐2B cells were transfected with 20 nM of locked nucleic acid (LNA) antisense oligonucleotides (Ambion) against *miR‐101* using Lipofectamine™ RNAiMax (Invitrogen). *miR‐20a* scramble LNA^14^ was utilized as the control oligonucleotide.

### 2.3 Plasmid construction and isolation of stable clones

BEAS‐2B cells were used for transfection of pcDNA3‐HA‐CERS6^15^ and then selected with 1 mM G418 (Nacalai Tesque) to establish bulk stable clones. For SK‐LC‐5 and RERF‐LC‐AI, *CERS6* was cloned into the pLenti 7.3/V5‐DEST Gateway vector (Invitrogen). Two days after lentiviral infection, green fluorescent protein‐positive cells were sorted using FACS Aria2 (BD). In some experiments, 2 short hairpin RNAs (shRNAs) with independent sequences against *CERS6* (shCERS6‐2 and shCERS6‐3, Table S1) were used.

### 2.4 *In vitro* motility, scratch and invasion assays

Motility and invasion assays were performed in vitro using Cell Culture Inserts (Transparent PET membrane, 24 wells, pore size 8 µm, BD Falcon). After cells were cultured in RPMI supplemented with 5% FBS overnight, the medium was replaced with RPMI containing N2 supplement (GIBCI‐BRL) and 20 ng/ml EGF to minimize ceramide uptake from the culture medium. Two days later, cells were collected using 5 mM EDTA in PBS, resuspended in RPMI supplemented with 0.1% FBS, seeded at a cell density of 1 × 10^5^ into the upper chambers and incubated for 16‐24 hours. For a scratch assay, LNM35 cells were plated in 6‐well plates overnight and a single linear line was created with a 200‐µl pipette tip.

### 2.5 Immunocytochemistry and immunohistochemistry

After cells were cultured in RPMI supplemented with 5% FBS overnight, the medium was replaced with RPMI containing N2 supplement. Two days later, the medium was replaced with RPMI supplemented with 10% FBS and left for 12‐16 hours. Cells were fixed and stained as previously described.^16^, ^17^ For immunohistochemistry, after antigen retrieval following microwave oven heating treatment, formalin‐fixed paraffin sections were subjected to immunoperoxidase assays using an avidin‐biotin peroxidase complex method.

### 2.6 Cell viability assays

Cells were plated at a density of 2 × 10^4^ cells/ml and cultured in RPMI supplemented with 5% FBS overnight. After siRNA treatment, the cells were further cultured in RPMI containing N2 supplement and 20 ng/ml EGF for 48 hours. Viable cells were measured in triplicate using TetraColor One (Seikagaku) with reference to the viability of mock‐treated cells.

### 2.7 Dual‐luciferase reporter assay

The 520‐bp region of the *CERS6* 3’ UTR segment was amplified by PCR using primers with the *Xba*I/*Apa*I site (Table S1) and ligated into a pGL3 vector (Promega). Mutations were introduced to the putative *miR‐101* binding site (Table S1) using a site‐directed mutagenesis method. Cell cultures, transfection and luciferase reporter assays were performed as previously described.^18^

### 2.8 Mice

Experimental metastasis assays using the A549 and LNM35 cancer cell lines were performed. Forty‐eight hours after cells were treated with mock, siCTRL or siCERS6‐1, A549 cells at 1 × 10^6^ or LNM35 cells at 3 × 10^6^ in 0.2 ml of RPMI‐1640 medium were injected into the tail vein of 6‐week‐old male nude mice (n = 8‐14). At 3 (A549) or 5 (LNM35) weeks after injection, the mice were killed and lung metastasis foci were assessed. When a mouse showed a poor condition, assessment was performed earlier.

### 2.9 Mass spectrometric analyses

After adding d18:1/C17:0‐ceramide (Avanti Polar Lipids) as the internal standard, the lipid fraction was extracted using the Bligh‐Dyer extraction method. Ceramide analyses were performed using high‐performance liquid chromatography (Shimadzu) with a 320 LC/MS/MS triple quadrupole tandem mass spectrometer (Agilent Technologies) or Acquity Ultra Performance LC (Waters) with 4000 QTRAP LC/MS/MS device (ABSciex). Mass spectrometry was performed in positive ion mode with an electrospray ionization source. For LC/MS/MS analyses, chromatographic separations were done in gradient mode using a conventional ODS column (Cadenza CW‐C18, 150 × 2 mm).^19^

### 2.10 RAC1 activation assay

LNM35 cells (4.5 × 10^6^) were used for transfection of 5 nM siCERS6‐1 or negative control siRNA using lipofectamine RNAiMax. For overexpression assays, RERF‐LC‐AI cells (6 × 10^5^) were used. After culturing both cell types for 6 hours in RPMI containing 5% FBS, the medium was replaced with RPMI1640 containing an N2 supplement (GIBCO‐BRL) and the culture was continued for 2 days, followed by serum stimulation using RPMI1640 with 10% FBS for 16 hours. RAC1 activation assays were performed using a Rac1/Cdc42 Activation Assay Kit (Millipore). Briefly, cells were harvested and dissolved in MLB (25 mM HEPES, pH 7.5, 150 mM NaCl, 1% Igepal CA‐630, 10 mM MgCl_2_, 1 mM EDTA, 10% glycerol) and then centrifuged at 9570 ×*g* for 15 seconds. Thereafter, PAK‐1 PBD agarose beads were incubated with each supernatant at 4°C for 1.5 hours and then washed 3 times with MLB, and binding proteins were analysed using Western blotting.

### 2.11 Ethics approval

Requisite approval from the review board of Nagoya University Graduate School of Medicine, Nagoya, Japan, and written informed consent from the patients were obtained prior to obtaining patient samples. The animal experiments were also approved by the review board of Nagoya University Graduate School of Medicine.

### 2.12 Statistical analysis

In the figure legends, the use of statistics in the experiments is indicated. A two‐tailed t test or Fisher's exact test was used as indicated. A log‐rank test was used for Kaplan‐Meier analyses. *P* values lower than 5% regarded as significant.

## 3 RESULTS

### 3.1 CERS6 overexpression and correlation with clinical outcome in NSCLC cases

**FIGURE 1:**
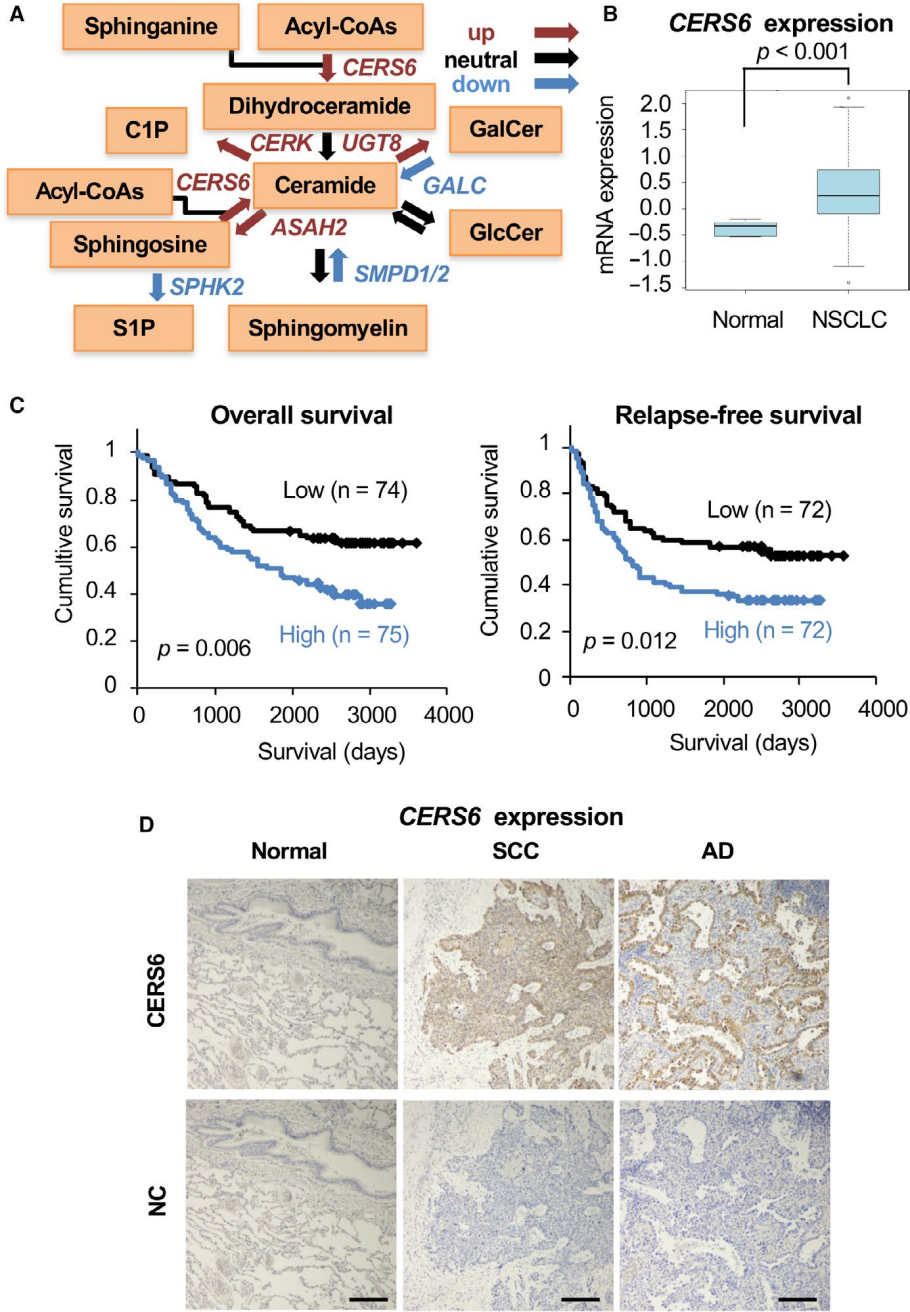
Overexpression of *CERS6* and correlation with clinical outcome in lung cancer. A, Metabolic pathway of ceramide and sphingolipid genes. Genes with up‐, neutral‐ and down‐regulation are shown by red, black and blue arrows, respectively (see Table S2). B, mRNA expression analysis of *CERS6* in normal tissues and NSCLC (5 normal lung mixtures and 149 NSCLC cases).^8^ A two‐tailed t test was used to determine *P* values. C, Overall survival and relapse‐free survival curves (Kaplan‐Meier analysis).^8^ Patients were classified into high and low groups using the median value of *CERS6* expression as the threshold. Cases without clinical information were not included. A log‐rank test was used to determine *P* values. D, Lung squamous cell carcinoma (SCC), adenocarcinoma (AD) and normal lung tissue (Normal) were used for immunohistochemical analysis of CERS6. NC, no primary antibody; bar = 0.2 mm.

We compared expression profiles of the ceramide metabolic pathway genes between NSCLC and normal lung tissues (Figure 1A, Table S2). Among the examined genes, the expression of *CERS6* was significantly elevated in NSCLC (Figure 1B) and also shown to be associated with poor patient prognosis (Figure 1C) as well as lymph node metastasis (Table 1). Analysis of another publicly available lung cancer data set showed similar results, with expression levels of *CERS6* well associated with prognosis (Figure S1). In accordance with the mRNA expression data, adeno and squamous cell carcinoma specimens showed obvious CERS6 protein expression, whereas the control tissues did not (Figure 1D, Table S3). Interestingly, other *CERS* genes including *CERS5*, a gene product with a substrate specificity similar to that of *CERS6*,^15^ did not show a significant correlation or expression pattern (Table S2, Figure S2).

**TABLE 1:** Association between CERS6 (AA758229_r_271) expression levels and clinical characteristics

		pT = 1	pT = 2	pT = 3	pT = 4	Total	*P*‐value
*CERS6*	High	20	41	8	6	75	0.155
	Low	30	32	10	2	74	

		pN = 0	pN = 1	pN = 2	pN = 3	Total	*P*‐value^1^
*CERS6*	High	40	15	18	2	75	0.039
	Low	53	6	15	0	74	

		pStage = 1	pStage = 2	pStage = 3		Total	*P*‐value
*CERS6*	High	34	15	26		75	0.235
	Low	44	11	19		74	

		*EGFR* = WT	*EGFR* = Mut			Total	*P*‐value
*CERS6*	High	61	14			75	0.247
	Low	54	20			74	

		*KRAS* = WT	*KRAS* = Mut			Total	*P*‐value
*CERS6*	High	69	6			75	1
	Low	68	6			74	

		*TP53* = WT	*TP53* = Mut			Total	*P*‐value
*CERS6*	High	35	40			75	0.071
	Low	46	28			74	

Note

1 *P‐values* for other CERS genes and pN status are 0.930 (CERS1, A_23_P209098), 0.217 (CERS1, A_23_P79032), 0.144 (CERS2, A_23_P63009), 1 (CERS3, A_23_P77151), 0.482 (CERS4, A_23_P153867), and 0.321 (CERS5_A_23_P76515).

### 3.2 CERS6 directly targeted by *miR‐101*

**FIGURE 2:**
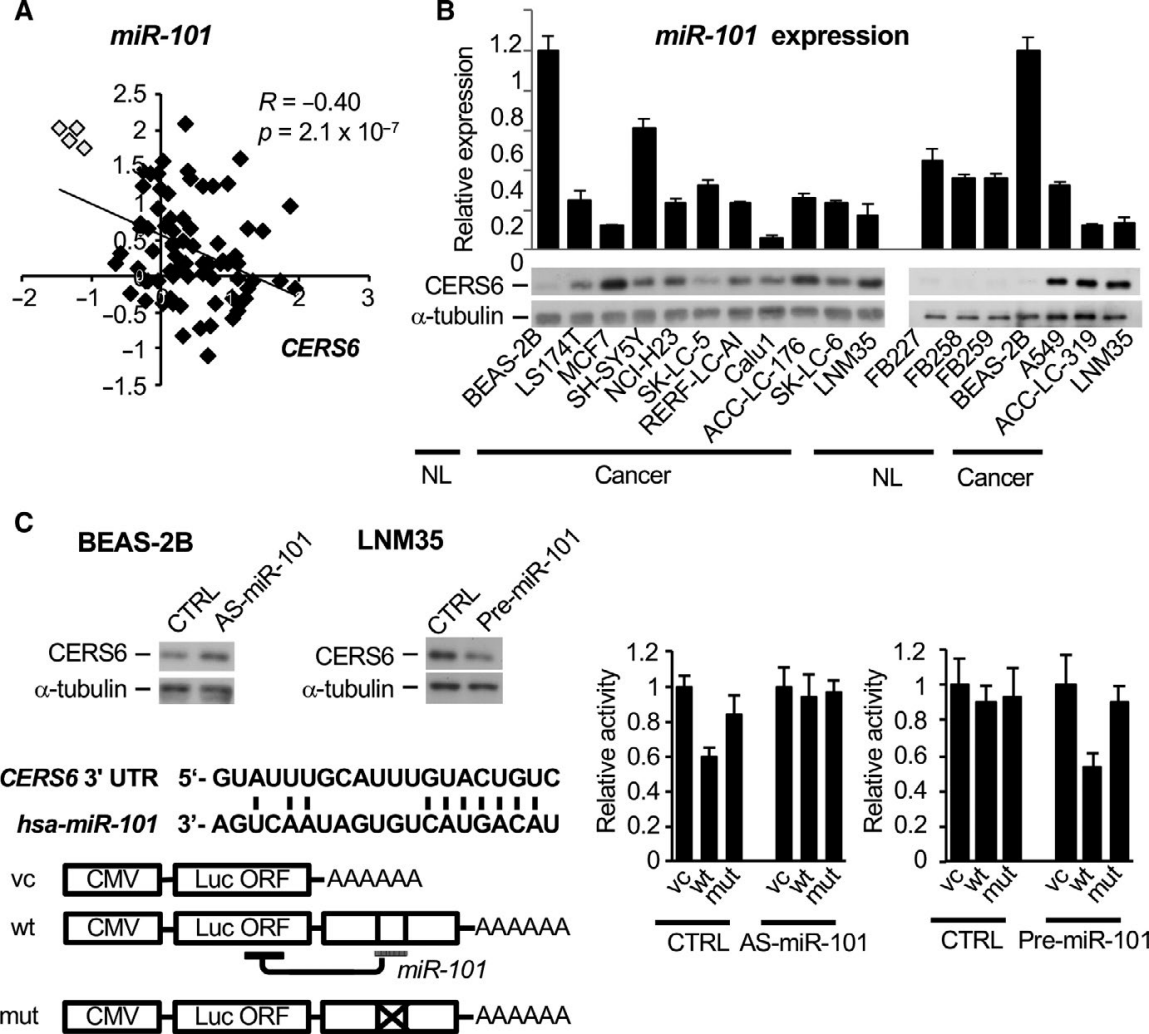
*miR‐101* controls *CERS6* expression. A, Expression levels of *miR‐101* and *CERS6* were examined in a cohort of adenocarcinoma (filled rectangle) and normal specimens (open rectangle).^32^ A two‐tailed t test was used to determine *P* values. B, *miR‐101* (top, relative to BEAS‐2B) and CERS6 (bottom) expression levels in a cancer cell panel, normal human lung fibroblasts (FB227, FB258, FB259) and normal human bronchial epithelial cells (BEAS‐2B). C, CERS6 protein expression was examined using Western blotting analysis (left, top). Schematic illustration of 3' untranslated region (UTR) of *CERS6* mRNA (left, bottom). Grey bar represents a putative binding site of *miR‐101* predicted by TargetScan. After *miR‐101* was silenced in BEAS‐2B cells or *miR‐101* was overexpressed in LNM35 cells, luciferase reporter analysis was performed (right). CTRL, AS‐miR‐101 and pre‐miR‐101 were used as a negative control, *miR‐101* antisense and *miR‐101* precursor, respectively (n = 3, mean ± SD). Experiments were replicated and similar results obtained.

MicroRNAs (miRNAs) are frequently deregulated in cancer.^20^ To elucidate the regulation mechanism of CERS6 expression, prediction algorithms were employed to nominate miRNAs putatively targeting *CERS6*. Among the predicted genes, 5 of the 6 examined miRNAs were detected in clinical samples (Figure 2A, Figure S3). Of those, *miR‐101* alone showed a high level of expression in normal tissues along with an obvious negative correlation with *CERS6*. Subsequent analysis showed that a high level of *miR‐101* expression was detected in the normal cell lines, while similar or lower expression levels were seen in the cancer cell lines (Figure 2B), whereas other miRNAs did not show such patterns (Figure S4).

We further examined whether *miR‐101* directly targeted *CERS6*. After knocking down *miR‐101* using an antisense RNA, CERS6 expression was moderately increased in the *miR‐101‐*high and CERS6‐low cell line BEAS‐2B (Figure 2B and C left‐top). We then constructed reporter plasmids for luciferase analysis (Figure 2C left‐bottom), and those results showed that the wild‐type *CERS6* 3’UTR, but not the mutant 3′ UTR sequence, suppressed luciferase activity (Figure 2C right). Transfection of antisense miR‐101 largely cancelled the suppressive effect of wild‐type 3′ UTR. An additional experiment performed using LNM35, a *miR‐101‐*low and CERS6‐high cell line, and an inverse pattern was obtained. These results strongly suggest that *CERS6* is directly targeted by *miR‐101*.

### 3.3 CERS6 promotes lung cancer migration and metastasis

**FIGURE 3:**
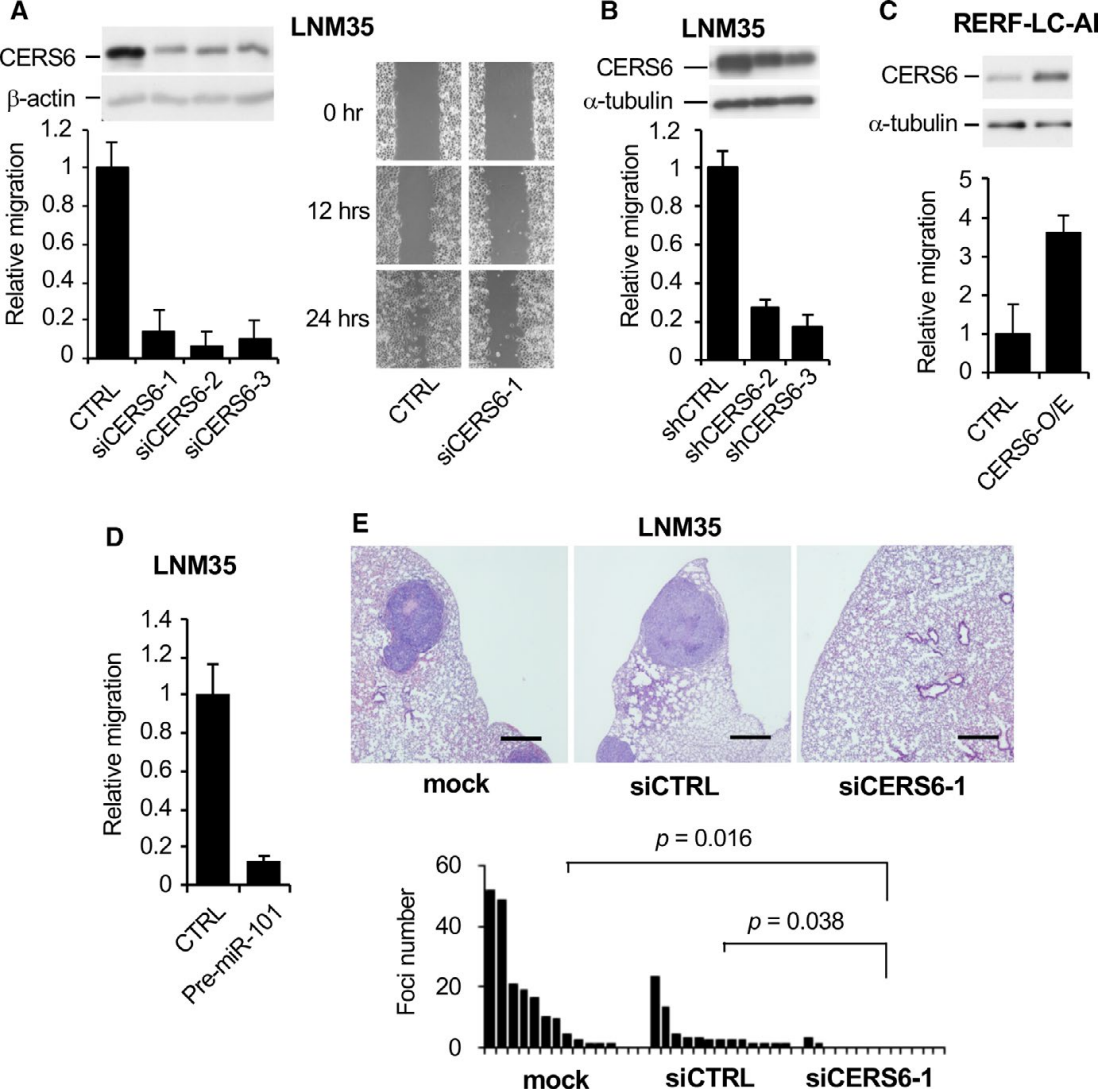
Lung cancer metastasis may be promoted by CERS6. A, Motility (left) and scratch (right) assays. CTRL, negative control siRNA; siCERS6‐1, −2 and −3 independent siRNAs targeting *CERS6*. Left, LNM35 cells with relative values to CTRL experiment are plotted (n = 4, mean ± SD). An immunoblot panel using an anti‐CERS6 antibody is shown on top. The experiments were replicated and similar results were obtained. B, Effects of shRNAs on the motility of LNM35 cells. shCTRL, vector control shRNA; shCERS6‐2 and shCERS6‐3, 2 independent shRNAs targeting CERS6. Relative values to CTRL experiments are plotted (n = 3 or 4, mean ± SD). Results of immunoblot analysis with an anti‐CERS6 antibody are shown on top. C, Effect of CERS6 overexpression (CERS6‐O/E, bulk clones) on motility of RERF‐LC‐AI. Relative values to those in the CTRL experiment are plotted (n = 6, mean ± SD). An immunoblot panel with anti‐CERS6 is shown on top. D, Effect of pre‐miR‐101 on motility of LNM35 cells. CTRL, negative control; Pre‐miR‐101, precursor of *miR‐101* (n = 4, mean ± SD). E, Five weeks after LNM35 cells were injected into the tail vein, mice were analysed for lung metastasis. A t test was used to determine *P* values. Representative microscopic images are shown on top. Bar = 0.5 mm. Quantified metastatic foci are also shown. Results of replicated experiment using A549 are shown in Figure S5D.

In order to analyse the functions of CERS6 in cancer, we treated LNM35 with small interfering or hairpin RNAs against *CERS6* (siCERS6/shCERS6). Both siRNAs and shRNAs significantly suppressed cell migration in motility, scratch and invasion assays (Figure 3A and B, and S5A). In contrast, under the CERS6 overexpression migration was stimulated in RERF‐LC‐AI, a cell line with a low level of CERS6 expression (Figure 3C). Similar results were obtained in examinations of other lung cancer cell lines, as knockdown was associated with decreased migration activities in A549 and ACC‐LC‐319, and overexpression stimulated that in SK‐LC‐5 (Figures S5B and C). Furthermore, CERS6 down‐regulation by *miR‐101* expression significantly attenuated LNM35 cell motility (Figure 3D).

The effect of CERS6 down‐regulation was also observed in vivo. When LNM35 or A549 cells were treated with siCERS6 and injected into mouse tail veins, significantly reduced metastatic activity was observed (Figure 3E, Figure S5D).

Based on these results, we suggest that CERS6 is a protein that promotes cancer cell migration and metastasis. CERS6 reduction in both LNM35 and ACC‐LC‐319 cells showed marginal effects on cell proliferation in vitro (Figures S6A and B); thus, CERS6 may not promote metastatic functions as a simple outcome of cell survival or increased proliferation.

In addition to CERS6, we also noted that other CERS family proteins had effects on cell migration activity in vitro (Figure S6C). Those were not analysed further, because their clinical significance has not been demonstrated (Tables 1, Table S2).

### 3.4 CERS6 and its enzymatic product ceramide required for lamellipodia formation

**FIGURE 4:**
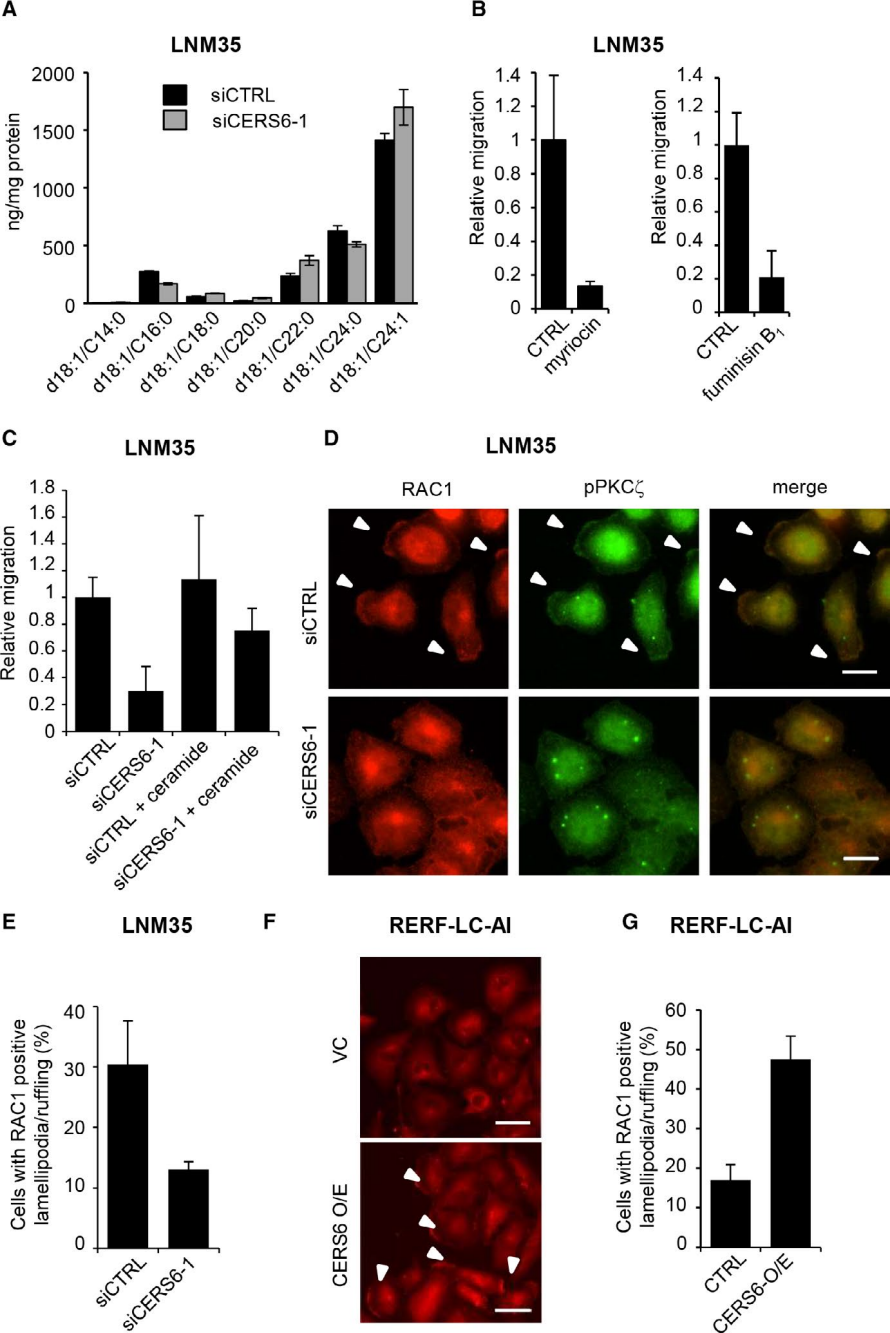
Ceramide synthesis may contribute to migration activity and lamellipodia formation. A, Ceramide amounts were quantitated in LNM35 cells (n = 3). The experiment was replicated, and similar results were obtained. B, Migration assays were performed with myriocin (100 nM, n = 4, mean ± SD) or fumonisin B_1_ in LNM35 cells (20 µM, n = 3, mean ± SD). Myriocin and fumonisin B_1_ experiments were replicated and triplicated, respectively, and similar results were obtained. C, After treatment of LNM35 cells with siCERS6‐1, migration activity was determined with C16 ceramide (1 µM) (n = 4, mean ± SD). The experiment was replicated, and similar results were obtained. D, LNM35 cells were starved and stimulated by serum for 12 hours and then subjected to immunocytochemistry using anti‐RAC1 and anti‐pPKCζ antibodies. Bar = 10 µm. E, In groups of 100 cells or more, those with RAC1‐positive lamellipodia were counted and the results plotted (percentage, mean ± SD). F, RERF‐LC‐AI cells were starved and stimulated for 12 hours and then subjected to immunocytochemistry using an anti‐RAC1 antibody. Bar = 50 µm. Representative image of triplicate experiments is shown. G, In groups of 100 cells or more, those with RAC1‐positive lamellipodia were counted and the results plotted (percentage, mean ± SD).

CERS6 is an enzyme that produces C16 ceramide. In the present experiments, CERS6 knockdown consistently reduced the amount of C16 ceramide, which was associated with a compensatory increase in ceramides with longer acyl chains (Figure 4A), probably due to the ‘inter‐regulation effect’ reported elsewhere.^21^ To examine whether ceramides stimulate cancer cell migration, LNM35 cells were treated with myriocin, which inhibits upstream serine palmitoyltransferase, or fumonisin B_1_, an inhibitor of CERS family proteins. In accordance with the idea that cell migration requires cellular ceramide synthesis, migration activity was significantly impaired (Figure 4B). Furthermore, addition of C16 ceramide to the culture medium partially recovered migration activity when LNM35 cells were treated with siCERS6 (Figure 4C). Taken together, CERS6 may stimulate cancer cell migration by enzymatic activity to synthesize C16 ceramide.

Cell migration is associated with a cell‐structural alteration, that is, lamellipodia/ruffling (hereafter lamellipodia) formation. Lamellipodia formation is induced by PKCζ activation, which results in a complex formation with RAC1.^22^ When cells were treated with a PKCζ pseudo‐substrate, migration activity was inhibited (Figure S7A). To determine whether C16 ceramide is involved in this pathway, we starved and then stimulated LNM35 cells with serum. Lamellipodia structures were seen in approximately one‐third of the cells, and CERS6 knockdown reduced the frequency (Figure 4D and E, Figure S7B), while CERS6 overexpression in RERF‐LC‐AI showed a reciprocal pattern (Figure 4F and G).

**FIGURE 5:**
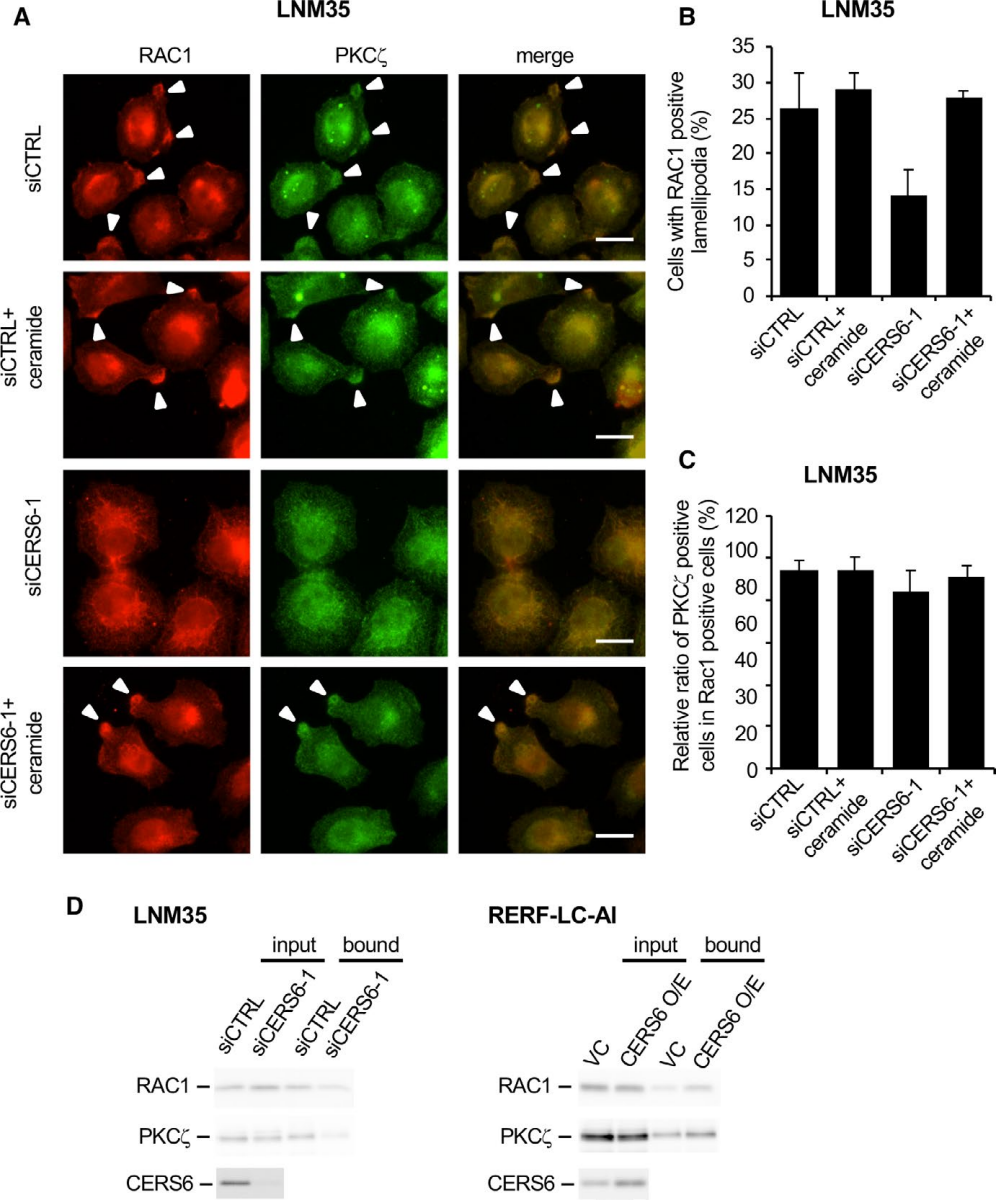
C16 ceramide is required for lamellipodia formation. A, LNM35 cells were starved and stimulated by serum for 12 hours and then subjected to immunocytochemistry using anti‐RAC1 and anti‐PKCζ antibodies. Results of experiments with C16 ceramide (1 µM) in culture medium are also shown. Bar = 20 µm. B, In groups of 100 cells or more, those with RAC1‐positive lamellipodia were counted and the results plotted. Values (mean ± SD) from triplicate experiments are shown. C, Among RAC1‐positive lamellipodia cells, the percentage of PKCζ‐positive cells was determined. Values (mean ± SD) from triplicate experiments are shown. D, LNM35 cells were treated with either siCTRL‐ or siCERS6‐1 and then analysed regarding the active RAC1 complex. For RELF‐LC‐AI cells, stable clones with either vector control (VC) or CERS6 overexpression (CERS6 O/E) were used. Input and bound fractions were subjected to Western blotting analyses of RAC1, PKCζ and CERS6. Experiments were replicated and similar results obtained.

Lamellipodia formation was considered to be dependent on the presence of C16 ceramide, because ectopic addition of C16 ceramide rescued this phenotype (Figure 5A and B

). In agreement with previous studies,^22^, ^23^, ^24^, ^25^ PKCζ, its phosphorylated form and ceramides were co‐localized in the lamellipodia structures (Figures 4D, 5A and C, Figure S7C).

The involvement of CERS6 in lamellipodia formation was further evaluated using a biochemical method (Figure 5D). Knockdown of CERS6 in LNM35 cells resulted in a decreased amount of active RAC1 protein, while in a reciprocal manner CERS6 overexpression in RERF‐LC‐AI cells resulted in elevation of that protein. Accordingly, PKCζ was co‐precipitated with active RAC1, with the amounts decreased in CERS6‐knockdown cells and increased in CERS6‐overexpressed cells.

Cell motility alteration is often associated with modulation of epithelial‐to‐mesenchymal transition (EMT). However, the expression levels of cellular E‐cadherin, vimentin and membrane TGFβ receptor 1 were only marginally altered in CERS6‐knockdown cells (Figure S8).

Based on these results, we suggest that CERS6 stimulates cancer cell migration through formation of a ceramide‐dependent lamellipodia structure.

## 4 DISCUSSION

Alterations of ceramide and CERS family protein levels have been reported in association with various types of cancer,^5^, ^6^, ^26^, ^27^ though their roles in the underlying pathogenesis have not been elucidated. Based on the present results, we propose that cancer cells are associated with *miR‐101* reduction and CERS6 overexpression, alterations that promote metastasis in lung cancer cases.

**FIGURE 6:**
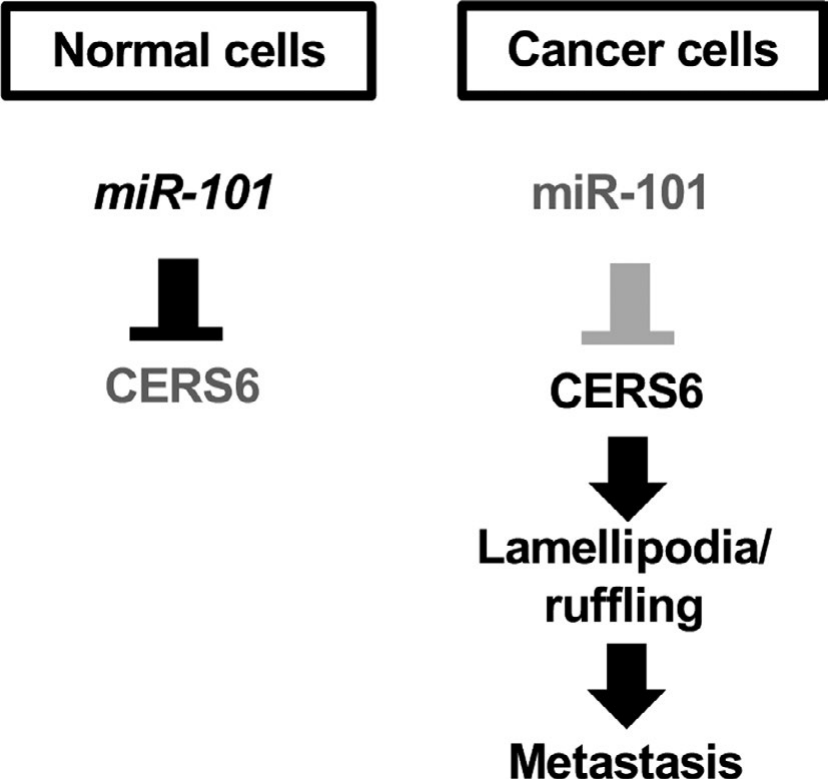
Schematic illustration of relationship between *miR‐101* and CERS6. In lung cancer, down‐regulation of *miR‐101* results in CERS6 up‐regulation and facilitates cell migration and metastasis

In relation to our proposal, cancer‐specific deletion of *miR‐101* has been reported,^28^, ^29^ while 37.3% of the cases in a previously presented lung adenocarcinoma data set exhibited loss of *miR‐101*.^30^ As for the present cohort, *CERS6* expression level was correlated with pN status, whereas the level of *miR‐101* expression was not (Tables 1, S4), possibly because *miR‐101* controls not only the expression of *CERS6* but also that of other tumour‐related genes. Taken together with results in prior studies, our findings are consistent with the idea that *miR‐101* has an onco‐suppressive function and also suggest activity of the *miR‐101*‐ CERS6 pathway contributes to lung cancer pathogenesis (Figure 6).

We also showed that CERS6 stimulated lamellipodia formation, which is essential for cancer cell metastasis.^22^ Thus, we consider that cancer cells show a metastatic phenotype as a result of CERS6 and C16 ceramide to produce a PKCζ and RAC1 complex.

Nevertheless, other possibilities cannot be denied, including the contributions of other sphingolipids to the phenotype. The present in vitro analysis showed that other CERS proteins have effects on cell migration activity (Figure S6C), suggesting that CERS6 and C16 ceramide are not the sole determinants for promotion of ceramide‐dependent cell migration. It should also be noted that in some cell lines, CERS6 was found to inhibit cell migration,^31^ suggesting that the *miR‐101*‐ CERS6 pathway does not explain all of these findings; thus, diverse pathways likely contribute to have effects on cancer cell phenotypes.

The present findings are considered to be useful for development of a drug to suppress CERS6 activity and metastasis. Given that CERS6 is not obviously expressed in normal lung tissues (Figure 1B and D), we believe that the *miR‐101*‐CERS6 pathway can be targeted, with potential benefits provided for affected patients.

## CONFLICTS OF INTEREST

The authors have no conflicts of interest to declare.

## AUTHORS’ CONTRIBUTION

Motoshi Suzuki: Funding acquisition (equal); Investigation (equal); Project administration (lead); Supervision (equal); Writing‐review & editing (equal). Ke Cao: Funding acquisition (supporting); Investigation (equal). Seiichi Kato: Investigation (equal). Naoki Mizutani: Investigation (equal). Kouji Tanaka: Investigation (equal). Chinatsu Arima: Investigation (equal). Mei Chee Tai: Investigation (equal). Norie Nakatani: Investigation (equal). Kiyoshi Yanagisawa: Methodology (equal). Toshiyuki Takeuchi: Investigation (equal). Hanxiao Shi: Investigation (equal). Yasuyoshi Mizutani: Investigation (equal). Atsuko Niimi: Investigation (equal). Tetsuo Taniguchi: Resources (equal). Takayuki Fukui: Resources (equal). Kohei Yokoi: Resources (equal). Keiko Wakahara: Resources (equal). Yoshinori Hasegawa: Resources (equal). Akira Satou: Investigation (equal). Yukiko Mizutani: Resources (equal). Soichiro Iwaki: Investigation (equal). Satoshi Fujii: Investigataion (equal). Keiko Tamiya‐Koizumi: Investigation (equal). Shuta Tomida: Investigation (equal). Takashi Murate: Investigation (equal). Mamoru Kyogashima: Methodology (equal). Takashi Takahashi: Funding acquisition (equal); Supervision (equal); Writing‐review & editing (equal).

## DATA SHARING

Data sharing is not applicable to this article as no new data were created or analysed in this study.

## Supporting information

Supplementary Material
