# Supplementary material for: CERS6 required for cell migration and metastasis in lung cancer
Source: J Cell Mol Med. 2020 Sep 9;24(20):11949–59. doi: 10.1111/jcmm.15817 (PMC7579715; doi:10.1111/jcmm.15817)
Supplement: Supplementary file 1 — Supplementary Material [file JCMM-24-11949-s001.pdf]

Supplementary Table 1

Experiment	Name	Sequence
Luciferase vector construction	CERS6 WT-3'UTR F	ATCGATCTGAGTCTAGACTCCTGCTCCATGGATGATT
	CERS6 WT-3'UTR R	ATCGATCGAGGGGCCCGCAATTTCAAAATGGGCACT
	CERS6 MUT-3'UTR F	CAGTATTTGCATTTGGTCTTAGAATATTA
	CERS6 MUT-3'UTR R	TAATATTCTAAGACCAAATGCAAATACTG
	CERS6 WT-3'UTR seqF	CTCCTGCTCCATGGATGATT
	CERS6 WT-3'UTR seqR	GCAATTTCAAAATGGGCACT
PCR	CERS1F	CTCATCGTCTCCTCCTACGC
	CERS1R	GGAACCAGAACCAGCTGAAG
	CERS2F	GTTTAACTACGCGGGATGGA
	CERS2R	GGCAGGATAGAGCTCCAGTG
	CERS5F	AAAATCCAATGCTGGTTTCG
	CERS5R	ACTGTCGGATGTCCCAGAAC
siRNA (forward sequence only)	CERS6 siRNA-1	r(AAGGUCUUCACUGCAAUUACA)dTdT
	CERS6 siRNA-2	r(CAACUGACCUUCACUACUA)dTdT
	CERS6 siRNA-3	r(GUGUGArCUCCUGUUUGUU)dTdT
	CERS1	r(GGUCCUGUAUGCCACCAGU)dTdT
	CERS2	r(GGAACAGAUCAUCCACCAU)dTdT
	CERS5	r(ACCCUGUGCACUCUGUAUU)dTdT
sh construct (forward sequence only)	CERS6 shRNA-2	GCAGGCCAATGGACCACAAATTCtcgaGAATTTGTGGTCCATTGGCC TGTTTTTT
	CERS6 shRNA-3	GCGGACGAACTAGGTGTTTAATCtcgaGATTAAACACCTAGTTCGTC CGTTTTTT

Supplementary Table 2

Gene	Probe	Med_NSCLC	Med_NL	T test	Logrank overall	Logrank relapse free	Pathway
CERS6	AA758229_r_271	0.25	-0.32	8.37E-06	0.006	0.012	de novo synthesis
SMPD2	A_23_P82159	0.21	0.52	6.58E-04	0.089	0.085	Sphingomyelin
DEGS1	A_23_P126186	-0.16	-0.07	0.144	0.073	0.05	de novo synthesis
SMPD3	A_23_P152186	0.12	0.11	0.375	0.09	0.358	Sphingomyelin
GBA2	A_23_P216536	0.002	0.13	0.018	0.177	0.012	HexCer (GlcCer)
CERS4	A_23_P153867	0.13	0.13	0.328	0.226	0.189	de novo synthesis
SPTLC1	A_23_P43326	0.29	0.49	1.40E-04	0.03	0.144	de novo synthesis
SMPD1	A_23_P203488	-0.25	1.46	2.45E-04	0.249	0.582	Sphingomyelin
PHCA	A_23_P203665	0.13	0.14	0.917	0.217	0.129	S1P
CERS1	A_23_P209098	-0.51	-0.39	0.365	0.316	0.459	de novo synthesis
TMEM23	A_23_P149791	0.36	0.39	0.464	0.222	0.508	Sphingomyelin
CERK	A_23_P211659	-0.02	-0.26	0.006	0.412	0.337	C1P
TMEM23	A_23_P115616	0.1	0.85	3.92E-04	0.448	0.543	Sphingomyelin
UGT8	A_23_P61346	-0.74	-1.52	2.77E-04	0.789	0.868	HexCer (GalCer)
CERS3	A_23_P77151	0.16	0.05	0.041	0.51	0.48	de novo synthesis
GALC	A_23_P25964	0.16	0.93	0.001	0.605	0.732	HexCer (GalCer)
GCS	A_23_P123645	-0.7	-0.2	0.087	0.407	0.739	HexCer (GlcCer)
TMEM23	AK026683_2141	0.07	0.21	0.072	0.264	0.712	Sphingomyelin
SMPD3	A_23_P163567	-0.93	-1.01	0.012	0.609	0.902	Sphingomyelin
SPTLC2	A_23_P3146	0.33	0.89	0.012	0.172	0.513	de novo synthesis
CERS1	A_23_P79032	0.86	1.33	2.15E-04	0.627	0.677	de novo synthesis
GBA3	A_23_P18672	-0.11	-0.33	0.017	0.917	0.457	HexCer (GlcCer)
SPHK1	A_23_P38106	-0.38	0.02	0.018	0.631	0.550	S1P
CERS2	A_23_P63009	-0.05	0.14	0.048	0.707	0.779	de novo synthesis
ASAH2	A_23_P161171	-0.35	-0.65	0.002	0.98	0.81	S1P
UGT8	A_23_P72747	-1.06	-2.31	0.003	0.29	0.314	HexCer (GalCer)
SPHK2	A_23_P208719	-0.46	-0.21	1.46E-05	0.987	0.447	S1P
CERS5	A_23_P76515	-0.08	-0.1	0.835	0.954	0.91	de novo synthesis

Altered ceramide metabolic gene expression in cancer tissues
Gene expression levels in ceramide metabolic pathways were compared among 149 NSCLC specimens and 5 normal lung mixtures. Genes with background levels or those without detection probes on the chip are not shown. *CERS6* expression level was associated with both overall and relapse-free survival. S1P, sphingosine-1-phosphate; C1P, ceramide-1-phosphate; HexCer, monohexosylceramide: GalCer, galactosylceramide; GlcCer, glucosylceramide.

Supplementary Table 3
Immunohistochemical expression of CERS6 in NSCLC and normal lung specimens

Lung Specimen(Lx)	Cancer	Normal lung	
		Type II pneumocyte	Pseudo-stratified ciliated epithelium
AD (L1)	2	0	0
AD (L2)	1	0	0
AD (L3)	2	0	0
AD (L4)	1	0	0
AD (L5)	1	0	0
AD (L6)	1	0	0
AD (L7)	2	0	1
AD (L8)	1	0	0
AD (L9)	2	0	0
AD (L10)	0	0	0
AD (L11)	1	0	0
AD (L12)	0	0	0
AD (L13)	1	0	0
AD (L14)	1	0	0
SCC (L15)	2	0	0
SCC (L16)	2	0	0
SCC (L17)	2	0	0
SCC (L18)	2	0	0
SCC (L19)	0	0	0
SCC (L20)	2	0	0
SCC (L21)	2	0	1
SCC (L22)	2	0	0
SCC (L23)	2	0	0
SCC (L24)	1	0	0
SCC (L25)	2	0	0
SCC (L26)	2	0	0
SCC (L27)	1	0	0

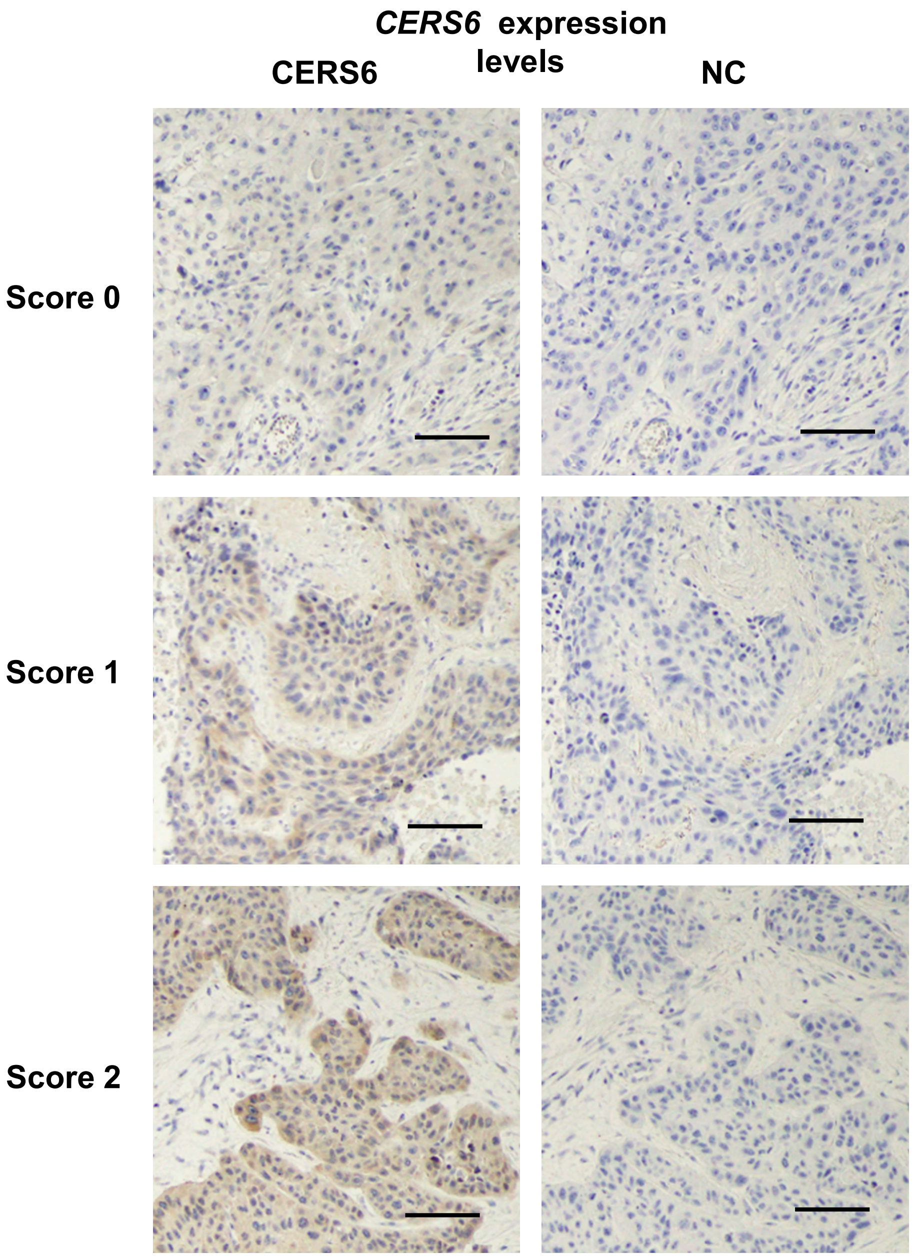

CERS6 protein highly expressed in NSCLC specimens.
Formalin-fixed paraffin sections were subjected to an immunoperoxidase study using an avidin-biotin peroxidase complex method. The CERS6 monoclonal antibody was used after antigen retrieval following microwave oven heating treatment. IHC stained slides were interpreted and scored on a scale ranging from 0 to 2, with samples with a staining score of 0 considered negative, and that of 1 and 2 weakly and strongly positive, respectively. Examples are shown in the right panels. AD, adenocarcinoma; SCC, squamous cell carcinoma. Bar, 0.2 mm.

Supplementary Table 4

Association between *miR-101* expression levels and clinical characteristics

	pT=1	pT=2	pT=3	pT=4	Total	p-value*
miR-101 high	22	31	4	5	62	0.384
low	17	33	9	3	62	
	pN=1	pN=2	pN=3	pN=4	Total	p-value*
miR-101 high	43	8	11	0	62	0.176
low	33	9	18	2	62	
	pStage=1	pStage=2	pStage=3		Total	p-value*
miR-101 high	39	5	18		62	0.033
low	26	14	22		62	
	EGFR=WT	EGFR=Mut			Total	p-value*
miR-101 high	40	22			62	0.001
low	56	6			62	
	KRAS=WT	KRAS=Mut			Total	p-value*
miR-101 high	55	7			62	0.763
low	57	5			62	
	TP53=WT	TP53=Mut			Total	p-value*
miR-101 high	41	21			62	0.046
low	29	33			62	

*p-values are determined by Fisher's exact test

Supplementary Fig. 1

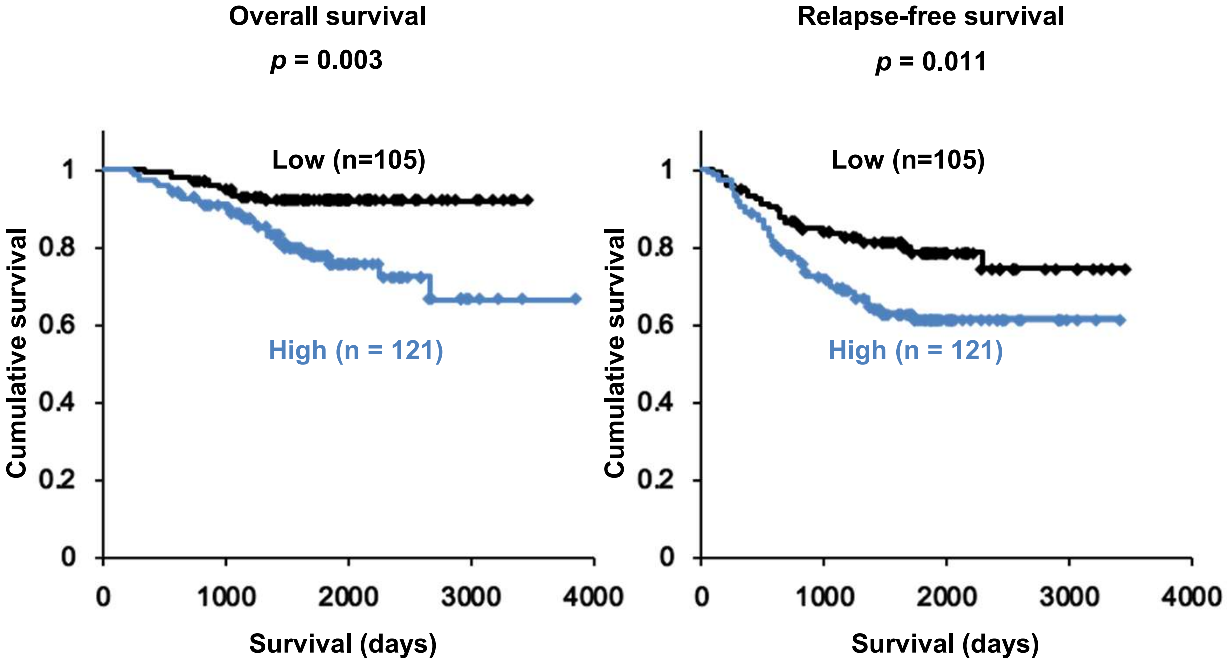

***CERS6* expression in breast cancer specimens.**
Kaplan-Meier analysis of mRNA expression level of *CERS6* and prognosis (data from Cancer Res 2012;72:100-111). The high and low groups were classified by *CERS6* expression level relative to the median value. Cases lacking clinical information were omitted from the analysis

Supplementary Fig. 2

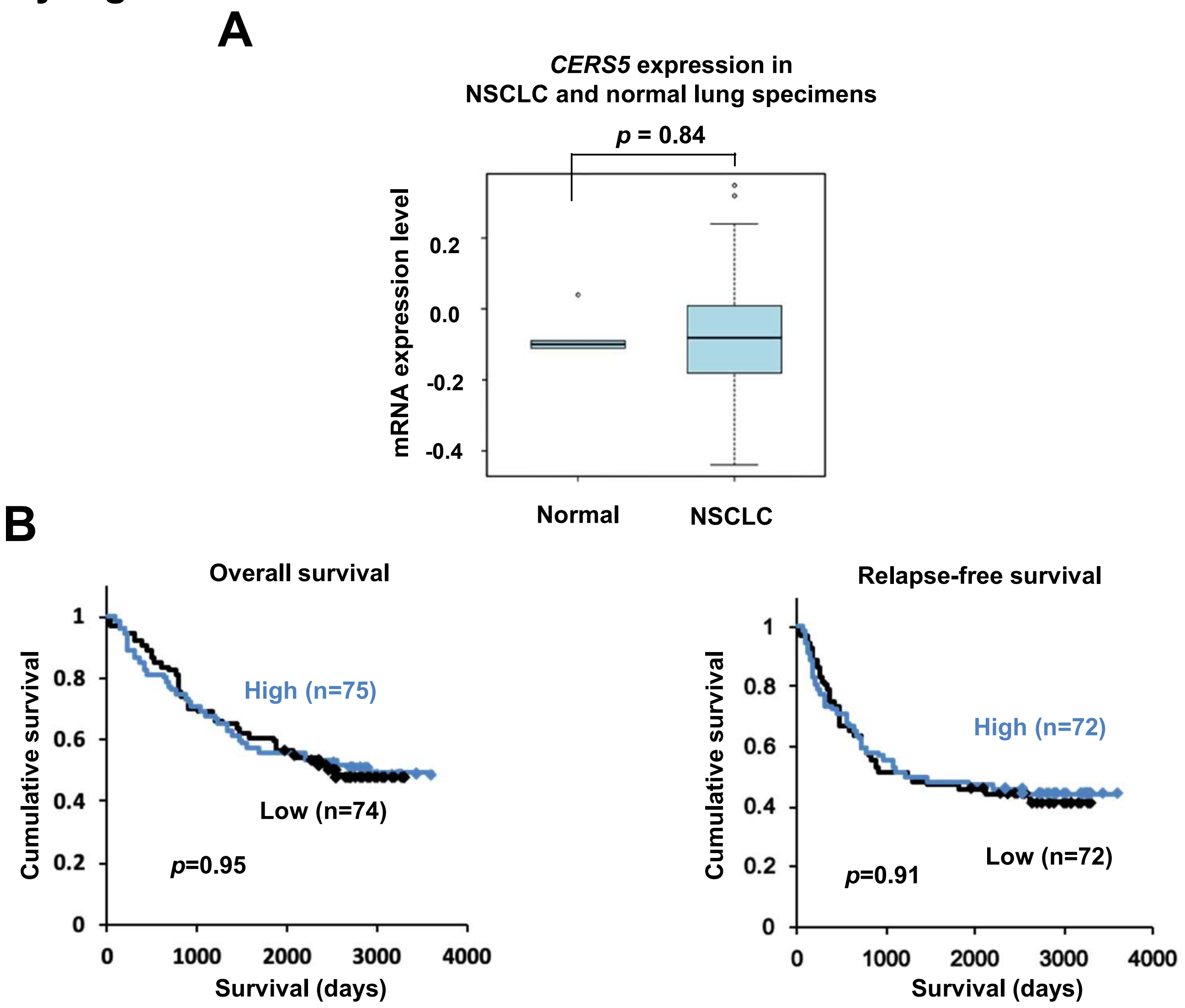

***CERS5* expression in NSCLC and normal specimens.**
(A) Box plot analysis of mRNA expression levels of *CERS5* in the 141 NSCLC and normal tissues. Normal, 5 normal lung mixtures; NSCLC, 141 cases. (B) Kaplan-Meier analysis showing overall survival (high and low, 74 and 75 cases, respectively) and relapse-free survival (high and low, 72 and 72 cases, respectively) curves in the 149 NSCLC cases. The high and low groups were classified based on *CERS5* expression levels relative to the median value.

Supplementary Fig. 3

A

Predicted miRNAs targeting *CERS6*

miRNA family	Context+ score	miRanda	Detection
hsa-miR-34a	-0.41	Y	Y
hsa-miR-217	-0.26	Y	N
hsa-miR-101	-0.23	Y	Y
hsa-miR-183	-0.17	Y	Y
hsa-miR-148a	-0.19	Y	Y
hsa-miR-200c	-0.10	Y	Y

B

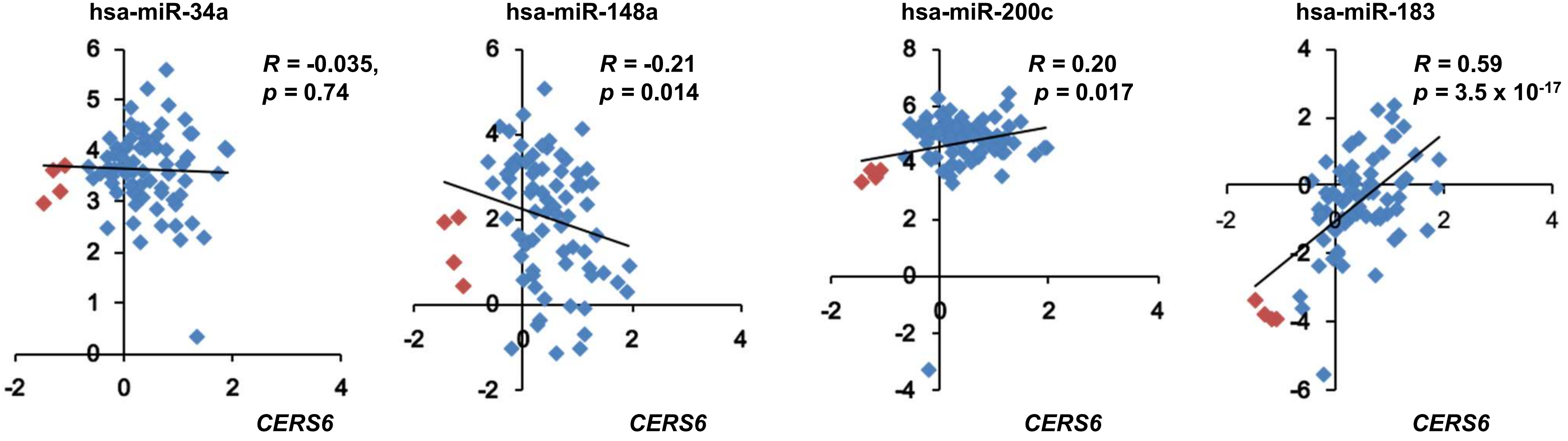

miRNA quantification using clinical specimens.

(A) Putative *CERS6*-targeting miRNAs were picked up using the prediction algorithms TargetScanHuman (Release 6.2) and miRanda. Conserved miRNAs were sorted according to the Context+ Score (TargetScanHuman). (B) Expression levels of the top 6 miRNAs in 79 adenocarcinoma (blue) and 4 normal (red) specimens were determined (Carcinogenesis 35; 2224-2231: 2014). In addition to miR-101 (Fig. 2), sufficient expression levels of miR-34a, miR-148a, miR-200c, miR-183, but not miR-217, were observed.

Supplementary Fig. 4

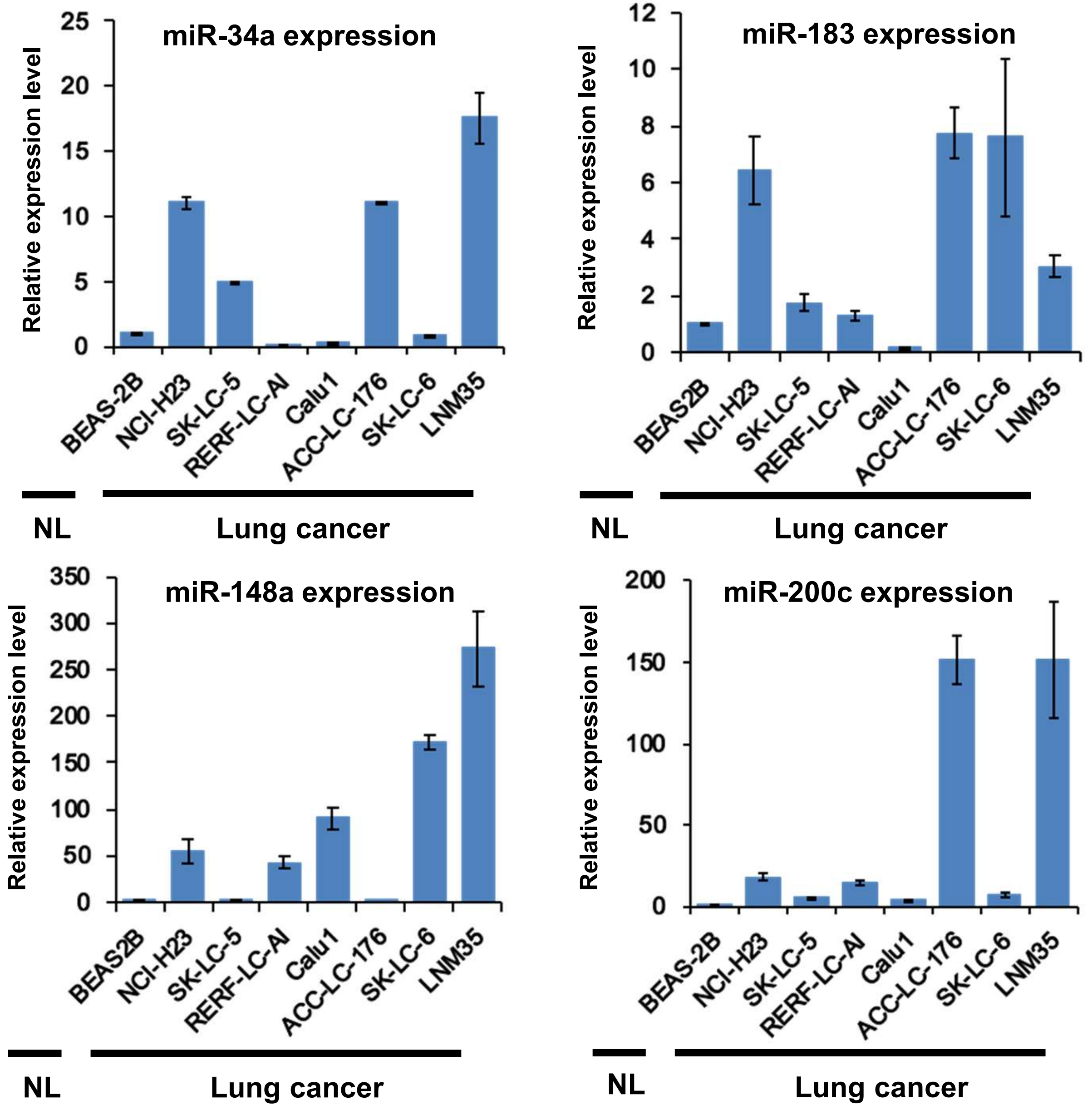

miRNA quantification using cell lines.

(A) Relative expression levels of miR-34a, miR-183, miR-148a, and miR-200c in a normal human bronchial epithelial cell line (NHBE) BEAS-2B and a panel of lung cancer cell lines were determined by quantitative RT-PCR. Bars, mean ± SD (n=3).

Supplementary Fig. 5

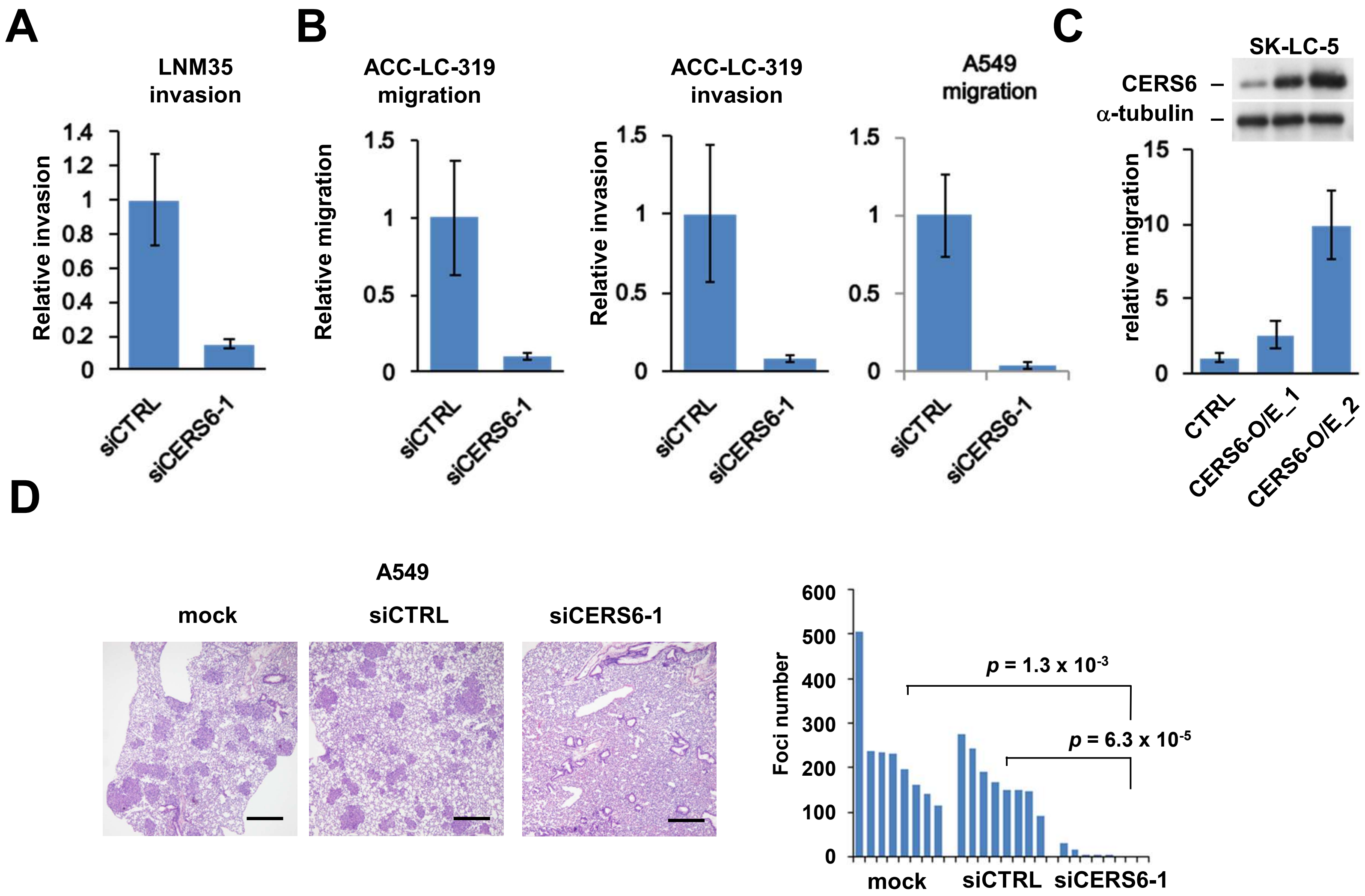

Knockdown of CERS6 suppresses lung cancer metastasis.

(A) Invasion assay to determine effects of CERS6 knockdown in LNM35 cells. CTRL, negative control siRNA; siCERS6-1, siRNA targeting *CERS6*. Bars, mean ± SD (n=3). (B) Migration and invasion assays were performed using ACC-LC-319 or A549 cells (n=4). (C) Migration assay to determine effects of CERS6 overexpression in SK-LC-5 cells. Two independent bulk clones were used. CERS6 expression levels are shown on top. (D) A549 cells were treated with mock, siCTRL, or siCERS6-1. Two days later, 1 x 10^6 cells were injected into tail veins (n=8). Three weeks after injection, the mice were euthanized to analyze lung metastasis. Left, representative lung samples are shown. Bar, 5 mm. Right, the number of metastasis sites was quantitated. *p* values were calculated using a two tailed t test.

Supplementary Fig. 6

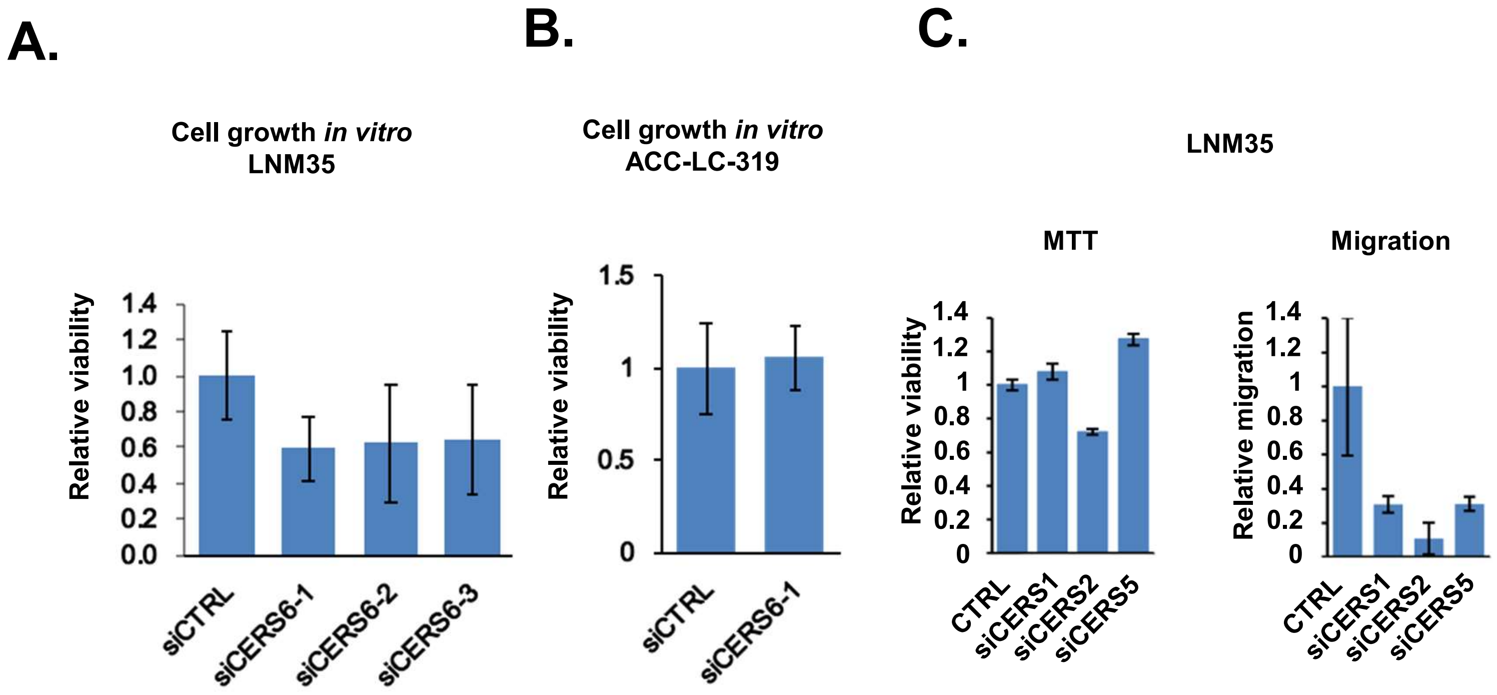

Knockdown of most CERS family genes showed only marginal effects on cell proliferation.
Five hours after LNM35 (A) or ACC-LC-319 (B) cells were treated with either 10 nM siCTRL or siCERS6-1~3, the culture medium was replaced with RPMI supplemented with EGF and N2 supplement. Cell viability was determined 48 hours after siRNA treatment. Bars, mean ± SD (n=6). (C) After treatment of LNM35 cells with the indicated siRNA, cell viability and migration activities were examined.

Supplementary Fig. 7

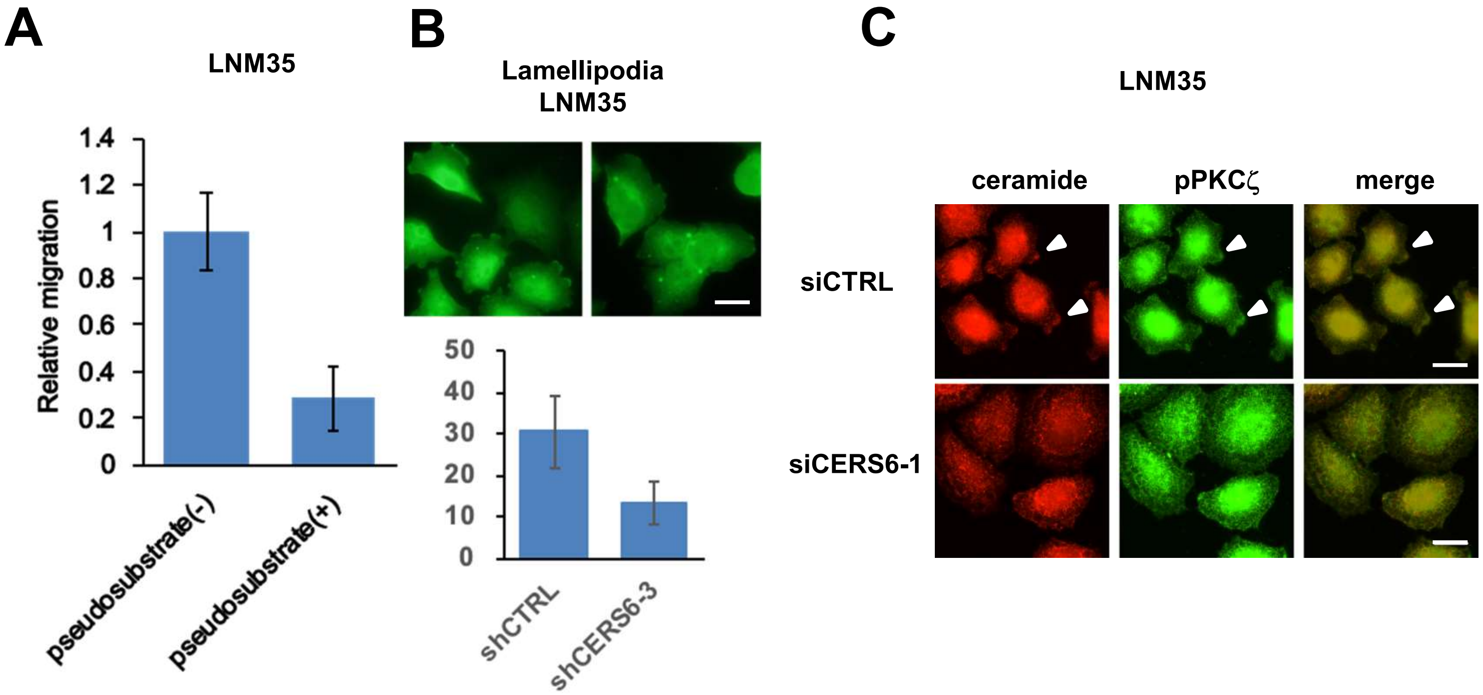

C16:0 ceramide may stimulate lamellipodia/ruffling formation through PKCζ activation.
(A) Migration assays were performed in the presence or absence of 1 μM PKCζ pseudo-substrate (Calbiochem). (B) Sixteen hours after serum stimulation, cells were fixed and stained by anti-RAC1 antibodies. Bar, 20 μm. (C) Twelve hours after serum stimulation, cells were fixed and stained by anti-ceramide and anti-pPKCζ antibodies. Bar, 20 μm.

Supplementary Fig. 8

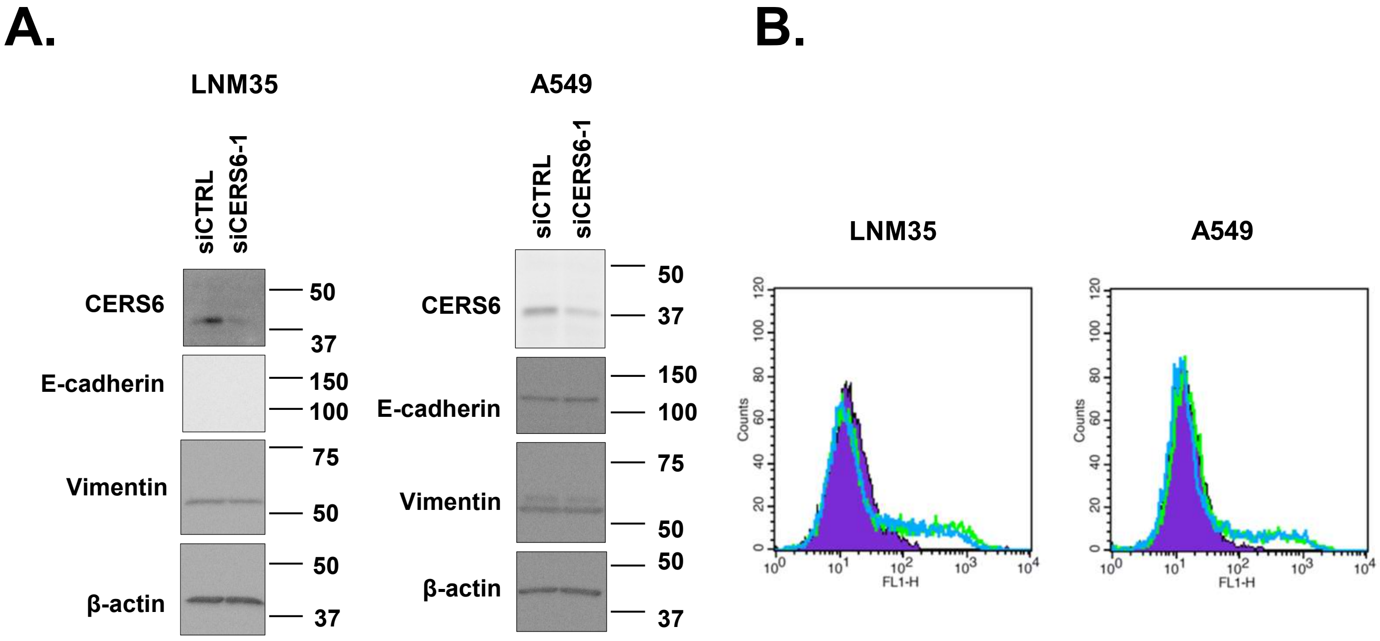

EMT assessment.

(A) Forty-eight hours after CERS6 knockdown, western blot analysis was performed with LNM35 and A549 cells using anti-E-cadherin (Arigo-Biolaboratories, ARG66195) and anti-vimentin (Arigo-Biolaboratories, ARG66199) antibodies. Experiments were replicated and similar results obtained. (B) In the LNM35 (left) and A549 (right) cell lines, membrane TGFβ receptor expression levels were quantitated by FCM analysis using anti-TGFβ receptor I (Abcam, ab235178) and Alexa Fluor 568 anti-rabbit IgG (Invitrogen, A21206) antibodies. Filled area, second Ab-only CTRL; green, siCTRL; blue, siCERS6-1). Experiments were replicated and similar results obtained.
